# Diurnal shifts of rumen fermentation and microbial profiles revealed circadian rhythms of rumen bacteria, methanogens, and protozoa under high-grain and high-forage diets

**DOI:** 10.3168/jdsc.2023-0526

**Published:** 2024-04-20

**Authors:** Yangyi Hao, Jianming Xia, Wei Wang, Yajing Wang, Zhijun Cao, Hongjian Yang, Linshu Jiang, Zhu Ma, Kangkang Chu, Shuang Wang, Le Luo Guan, Shengli Li

**Affiliations:** 1 State Key Laboratory of Animal Nutrition and Feeding, Beijing Engineering Technology Research Center of Raw Milk Quality and Safety Control, College of Animal Science and Technology, China Agricultural University, Beijing, 100193, China; 2 Department of Agricultural, Food and Nutritional Science, University of Alberta, Edmonton, AB T6G 2P5, Canada; 3 Beijing Key Laboratory of Dairy Cow Nutrition, College of Animal Science and Technology, Beijing University of Agriculture, Beijing 102206, China; 4 Beijing Dairy Cattle Center, Beijing 100192, China; 5 Faculty of Land and Food Systems, The University of British Columbia, Vancouver, British Columbia, V6T 1Z4 Canada

## Abstract

In this study, we investigated how the composition and population of rumen microbiota shifted in response to diurnal oscillations under 2 different diets (high grain vs. high forage). Five multiparous Holstein dairy cows with similar BW, DIM, and parity were enrolled in this study. The cows were fed high-grain (HG) diet for 21 d and then shifted to high-forage (HF) diet in the next 21 d (7-d washout and 14-d experimental period). During the experimental period, DMI and rumination activity were recorded, and rumen fluid was collected 8 times postfeeding every 6 h during the last 2 d of each dietary feeding period. The rumen microbial (bacterial, archaeal, and protozoal) population and composition were assessed using quantitative PCR and amplicon sequencing, respectively. The daily dynamic of measurements was assessed using the cosinor model. The associations between microbial taxa and rumen fermentation profiles were assessed using the linear mixed model, in which the cows were termed as random intercept effects. Daily rhythmicity was observed for DMI, rumination activity, and rumen fermentation profiles under both diets. Additionally, rumination time, rumen pH, and acetate/propionate ratio had a higher mesor (the average level of diurnal fluctuations) under HF diet than in HG diet. The amplitude (the distance between the peak and mesor) of DMI, rumen pH, ammonia nitrogen, and total volatile acid concentration were higher under the HG diet than in the HF diet. Although no significant diurnal oscillation was observed for the rumen microbial population, the relative abundance of 14 bacterial genera, 1 protozoal genus, and 2 archaeal species had significant diurnal oscillations under both HF and HG diets. Among them, the bacterial genera *Ruminococcus* and *Colidextribacter* had time at peak of rhythm within 0 to 12 h after feeding, which were also negatively associated with the rumen acetate/propionate ratio. The bacterial genus *Rikenellaceae_RC9_gut_group* had time at peak of rhythm within 12 to 24 h after feeding, which was also positively associated with the ruminal acetate/propionate ratio. Our study illustrated the daily dynamic on the rumen microbiota population and composition under different diets, and also identified the feeding-responsive rumen microbiota, highlighting that a more targeted approach is needed to manipulate rumen microbiota.

## Highlights

• The feed intake and rumination time had rhythmicity and were affected by diet.
• The relative abundance of rumen microbiota showed rhythmicity under different diets.
• Rhythmic *Rikenellaceae_RC9_gut_group* positively related to rumen acetate/propionate ratio.
• Rhythmic Ruminococcus and *Colidextribacter* negatively related to acetate/propionate ratio.

## Graphical Abstract

**Figure:**
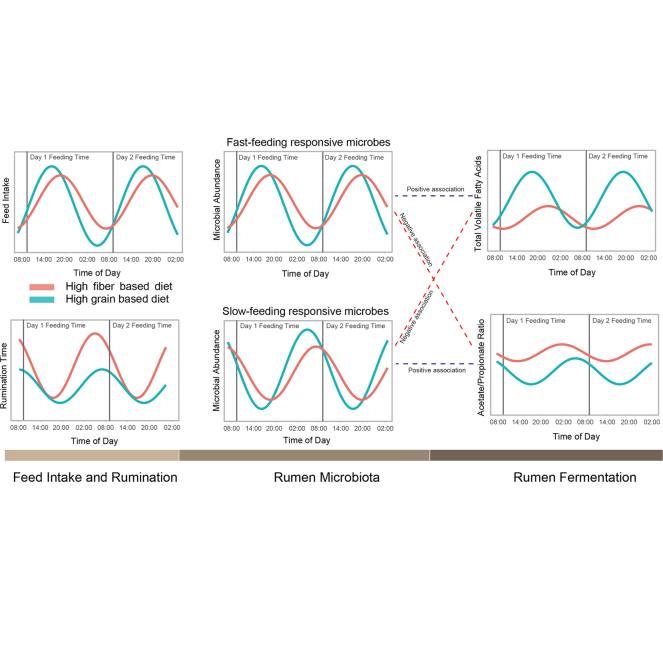
**Summary:** This study assessed the shift in feed intake, rumination time, rumen fermentation profiles, microbial population, and composition in response to diurnal oscillations under 2 different diets (high grain vs. high forage). The cows' feed intake and rumination had a significant rhythmicity. Additionally, the relative abundance of 14 bacterial genera, 1 protozoal genus, and 2 archaeal species had significant diurnal oscillations under both diets. However, the microbial taxa displaying contrasting daily dynamic patterns had different relationships with rumen total volatile fatty acid concentration and acetate/propionate ratio. These findings provide a fundamental understanding of the dynamic shift of rumen microbiota in response to diet and feeding time and its effects on rumen fermentation.

Rumen microbiota are complex and dynamic and their composition can be affected by many factors, resulting in altered functional outputs. Among them, dietary factors are the most studied and known to be one of the driving forces to change the rumen microbiota. High-grain (**HG**) and high-forage (**HF**) diets are the most common dietary types applied to cattle, and the different concentration-to-forage ratios in these diets can alter the composition and metabolites of the rumen bacterial community (Yi et al., 2022). A previous study indicates that the feeding rhythm can shape the diurnal oscillations of gut microbiota under the same diet in mice (Thaiss et al., 2014). Rumen bacterial, archaeal, and protozoal populations increase dramatically after feeding (Söllinger et al., 2018). In addition, feeding rhythm can lead to rumen fermentation profiles shifting through the feeding cycle (Ying et al., 2015; Salfer et al., 2021). However, it is known that different diet compositions can also change rumen fermentation (Ying et al., 2015). During ruminal fermentation, the accessible amorphous regions of the fiber are degraded rapidly, whereas the intricate crystalline areas of the fiber are degraded relatively slowly, suggesting that the functional microbial taxa also differ depending on the structure of feedstuff and affect the feed degradation patterns (Morais and Mizrahi, 2019a,b). Rumen microbiota are composed of various microbial taxa; it is still unknown whether diurnal oscillations of different functional microbial taxa are similar or not under distinct diet compositions.

Recent research has revealed that diurnal oscillations of gut microbiota were driven by feeding rhythm and nutrient substrates in pigs (Wang et al., 2023; Xu et al., 2023). It has been reported that jet lag can change food consumption rhythmicity, which further altered diurnal oscillations of gut microbiota in humans, and this gut dysbiosis is transferrable to germ-free mice upon fecal transplantation (Thaiss et al., 2014). In addition, the diurnal oscillations of colonic microbiota could be affected by nutrient substrates in the gut of pigs (Wang et al., 2023). In ruminants, a previous study reported diurnal oscillations of rumen bacterial and archaeal composition in lactating dairy cows under different forage structures, but the nutritional levels were similar (Shaani et al., 2018). However, this study did not assess the diurnal oscillations within the feeding cycle and it is unclear if protozoa could have similar diurnal oscillation patterns in the rumen because some bacteria and archaea have symbiotic relationships with protozoa (Newbold et al., 2015). Additionally, whether the diurnal oscillations of rumen microbiota can be shifted under different nutritional compositions of diet is still unknown. Taken together, we hypothesized that the diurnal oscillations of rumen microbiota can be different under distinct nutritional composition diets through the feeding cycle. We also speculated some rumen microbes could respond faster, whereas some of them respond more slowly to availability of nutrient and substrates. Such different microbial responses to the time postfeeding could affect the overall rumen fermentation profiles. Our objectives were to elucidate the diurnal oscillations of rumen microbiota population and composition under different nutritional composition diets, aiming to provide a better understanding to manipulate targeted rumen microbiota in the future.

Five Holstein dairy cows (during the transition from mid- to late-lactating period) with similar DIM (179 ± 22), parity (3 ± 0), and BW (639 ± 40, kg) were selected for this study. The main ingredients of the diet included whole-plant corn silage, soybean meal, steam-flaked corn, alfalfa, and oat grass. The proportions of these diet ingredients were adjusted and finally 2 diets were obtained: HG diet (18.08% CP, 30.40% NDF, and 25.61% starch, DM basis) and HF diet (14.52% CP, 39.38% NDF, and 19.26% starch, DM basis). Cows were fed HG diet for 21 d and then changed to HF diet for another 21 d (7-d washout and 14-d experimental period). During each feeding period, the diet was supplied once a day at 0800 h and cows had free access to water all the time. Cows were kept in a freestall barn and milked twice a day (0730 and 1930 h) following the herd standard operation protocol at the Beijing Nainiu Center Farm (Yanqing, Beijing, China). All procedures carried out in this study were approved by the China Agriculture University Laboratory Animal Welfare and Animal Experimental Ethical Faculty (protocol number: AW81102202–1-1).

Rumen fluid was collected using an oral gastric tube (Ancitech, Winnipeg, MB, Canada) every 6 h (in total 8 times) during the last 2 d of each feeding period, at 0800, 1400, 2000, and 0200 h for each day, respectively. Rumen fluid was separated into 2 parts: (1) stored at −20°C for the rumen fermentation profiling, and (2) kept −80°C for DNA extraction. Feed intake data were collected using Roughage Intake Control System (RFID, Zhenghong Company, Shanghai, China), which recorded DMI based on each cow's ID. Rumination activity was monitored using Neck-Mounted Accelerometer-Equipped Collars (Merck & Co. Inc., Rahway, NJ). Rumen fluid pH was measured immediately after rumen sample collection with a pH electrode (model pH B-4, Shanghai Chemical, Shanghai, China). Ammonia nitrogen (NH_3_-N) concentration was measured using the phenol-sodium hypochlorite colorimetry method on a spectrophotometer (721, INESA Analytical Instrument Co. Ltd., Shanghai, China) described by Broderick and Kang (1980). The VFA were measured using GC (6890 N, Agilent Technologies, Avondale, PA) following the methods described by Cao et al. (2008).

Total DNA was extracted from rumen fluid using the QIAmp Stool Mini Kit (Qiagen, Germany) following the manufacturer's instructions. The DNA quality was assessed with absorbance at 260 and 280 nm using ND-1000 spectrophotometer (NanoDrop Technologies, Wilmington, DE). The amplicons were generated using primers Ba9f (5′-GAGTTTGATCMTGGCTCAG-3′) and Ba515Rmod1 (5′-CCGCGGCKGCTGGCAC-3′); Ar915aF (5′-AGGAATTGGCGGGGGAGCAC-3′) and Ar1386R (5′-GCGGTGTGTGCAAGGAGC-3′); Reg841F (5′-GACTAGGGATTGGAGTGG-3′) and Reg1302R (5′-AATTGCAAAGATCTATCCC-3′) specific for bacteria, archaea, and protozoa, respectively (Henderson et al., 2015) and were subjected to sequencing using Illumina MiSeq (300 bp pair-end). The raw sequence data were processed and analyzed using QIIME2 (version 2022.2; Bolyen et al., 2019). Quality control, denoising, removal of chimeric sequences, and generation of amplicon sequencing variants (**ASV**) were performed using the QIIME2 plugin DADA2 (Callahan et al., 2016). Rumen microbiota taxonomic composition was obtained with the feature classifier command in QIIME2 using ASV against the SILVA database (version 138.1; Quast et al., 2013) for bacteria and protozoa, and the RIM-DB database (Seedorf et al., 2014) for archaea. Quantitative PCR (**qPCR**) was conducted to estimate the population of rumen microbial groups using respective universal primers for bacteria (U2-F: 5′-ACTCCTACGGGAGGCAG-3′; U2-R: 5′-GACTACCAGGGTATCTAATCC-3′) (Stevenson and Weimer, 2007), archaea (uniMet1-F: 5′-CCGGAGATGGAACCTGAGAC-3′; uniMet1-R: 5′-CGGTCTTGCCCAGCTCTTATTC-3′) (Zhou et al., 2009), and protozoa (SSU-316F: 5′-GCTTTCGWTGGTAGTGTATT-3′; SSU-539R: 5′-CTTGCCCTCYAATCGTWCT-3′) (Sylvester et al., 2004). The qPCR was conducted using SYBR Green chemistry (Fast SYBR Green Master Mix, Applied Biosystems) on the StepOnePlus Real-time PCR System (Applied Biosystems). Standard curves were made using serial dilutions of purified plasmid DNA for bacteria, archaea, and protozoa, respectively. The PCR programs and copy numbers of each standard curve and bacteria, archaea, and protozoa per milliliter of rumen fluid were calculated according to Zhou et al. (2009).

The daily patterns of feed intake, rumination time, rumen fermentation profiles, microbial population, and the relative abundance of microbial taxa were further evaluated by fitting to cosinor model and the difference between the 2 diet groups was analyzed with the “circacompare_mixed” function in ‘Circacompare' R package (v0.1.1; Parsons et al., 2020). Because there were no diurnal oscillations for microbial populations, they were further analyzed using the MIXED procedure (version 9.4, SAS Institute Inc., Cary, NC). Fixed effects included diet, time of the day, and their interaction. The cow ID and DIM were included as a random effect. Additionally, the microbial population of each time point between the 2 diets was also analyzed by paired *t*-test based on their normality and homogeneity of variance. Linear mixed-effects model (**LMM**) was performed using ‘lme4' R package (v1.8–42) (Bates et al., 2015) to assess the correlation between feeding-responsive microbiota and fermentation profiles, feed intake, and rumination, in which the cows were termed as random intercept effects. The correlation coefficient in the LMM represents the relationship between microbiota and fermentation profiles. Due to the different time of day and consecutive 2 d of sampling that could be autocorrelated, a nested autoregressive model was included to account for temporally correlated errors within the LMM. The *P*-value from the LMM was assessed by the Wald type II χ^2^ tests using the ‘car' R package (v3.1.1). Only correlation coefficients >0.4 and *P* < 0.05 were considered significant in this study. A *P*-value <0.05 indicates a significant difference for all of the analyses.

The cows' DMI and rumination had a circadian rhythm (*P* < 0.05) under both HG and HF diets. The peak of the DMI rhythm occurred during the daytime, and the peak of the rumination rhythm happened in the nighttime (Figure 1). There was no difference in the mesor of DMI between the HG and HF diets; however, cows had a greater (*P* < 0.001) mesor of rumination time under the HF diet compared with the HG diet. The amplitude of DMI under the HG diet was higher (*P* < 0.001) than that under the HF diet, and the peak time of DMI was also delayed (*P* = 0.003) under the HF diet compared with the HG diet. The amplitude and peak time of rumination time had no difference between the 2 diets. Rumen pH, NH_3_-N, TVFA, and acetate/propionate (**A/P**) ratio had a circadian rhythm under each diet (*P* < 0.05). The cows under the HG diet had a lower mesor of rumen pH and A/P ratio than that under HF diet (*P* < 0.01); however, the rumen NH_3_-N and TVFA concentrations under the HG diet had a greater mesor than those under the HF diet (*P* < 0.01). The rumen fermentation profiles under the HG diet had higher amplitudes of pH, NH_3_-N, and TVFA diurnal rhythm under the HG diet compared with the HF diet (*P* < 0.05), suggesting stronger diurnal oscillations under the HG diet. The amplitude and peak time of A/P ratio had no difference under the 2 diets.

**Figure 1:**
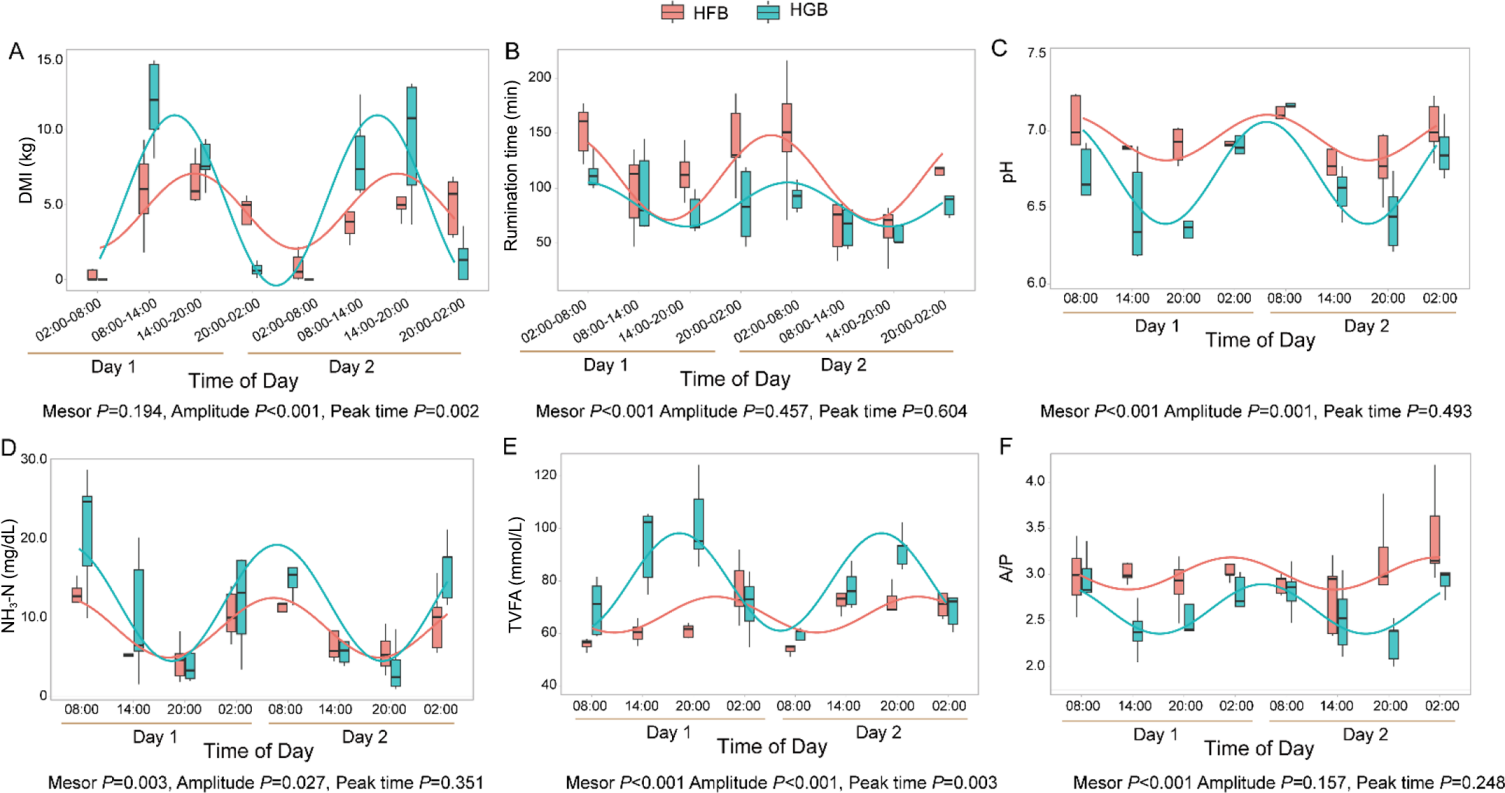
The daily pattern of DMI (A), rumination (B), and rumen pH (C), NH_3_-N (D), TVFA (E), and A/P (F) under HF and HG diets. Boxes represent the interquartile range (IQR) between the first and third quartiles, and the horizontal line inside the box represents the median. Whiskers represent the lowest and highest values within 1.5 times the IQR from the first and third quartiles, respectively. The lines represent the cosinor fitted curve. Mesor: the average level of the diurnal fluctuation. Amplitude: the distance between peak and mesor. Peak time: the time of the peak of rhythm.

No significant diurnal oscillation was observed for rumen bacterial, archaeal, and protozoal populations (Figure 2). There were also no significant effects of time and the interaction of time and diet on rumen bacterial, archaeal, and protozoal populations. Nevertheless, the protozoal population was higher (*P* < 0.001) under the HG diet compared with the HF diet. Additionally, the population of archaea and protozoa was greater (*P* < 0.001) at 0200 h of the second sampling day under HG diet than under HF diet. The relative abundance of 14 bacterial genera, archaeal species *Group12 sp-ISO4-H5* and *Methanobrevibacter ruminantium clade*, and protozoal genus *Isotricha* had a diurnal oscillation (*P* < 0.05) under both HG and HF diets. Among these microbes, *Lachnospiraceae_NK3A20_group*, *Ruminococcus*, *Muribaculaceae*, *F082*, *CAG-352*, *Treponema*, and *Colidextribacter* and archaeal species *Methanobrevibacter ruminantium clade* had their peak time in the range of 0 to 12 h after feeding (0800–2000 h), and they were defined as fast-feeding responsive (**FFR**). Bacterial genera *Izemoplasmatales*, *Lachnospiraceae_XPB1014_group*, *Pseudobutyrivibrio*, *UCG-002*, *Butyrivibrio*, *UCG-004*, *Rikenellaceae_RC9_gut_group*, protozoal genus *Isotricha*, and archaeal species *Group12 sp-ISO4-H5* also had their peak time in the range of 12 to 24 h after feeding (2000–0800 h), and they were classified into slow-feeding-responsive (**SFR**) taxa. The daily patterns of relative abundance of these FFR and SFR microbes are shown in Figure 2. Additionally, the mesor value in the rhythm of the relative abundance of *Colidextribacter*, *Lachnospiraceae_NK3A20_group*, *Ruminococcus*, *Izemoplasmatales*, and *UCG-002* was higher under the HG diet than under the HF diet, whereas the opposite trend was observed for the genus *Butyrivibrio*. The relative abundance of FFR taxa *Colidextribacter* (r = −0.43, *P* < 0.001) and *Ruminococcus* had a negative correlation (r = −0.47, *P* < 0.001) with A/P ratio, whereas the relative abundance of SFR taxa *Rikenellaceae_RC9_gut_group* had a positive correlation (r = 0.42, *P* < 0.001) with A/P ratio (Figure 3). Two microbial taxa had a significant correlation with TVFA and also had a negative correlation with pH: *Ruminococcus* (TVFA: r = 0.56, *P* < 0.001; pH: r = −0.52, *P* < 0.001) and *Rikenellaceae_RC9_gut_group* (TVFA: r = −0.47, *P* < 0.001; pH: r = 0.41, *P* < 0.001). Furthermore, the relative abundance of *Ruminococcus* was positively associated with starch intake correlation (r = 0.49, *P* < 0.001).

**Figure 2:**
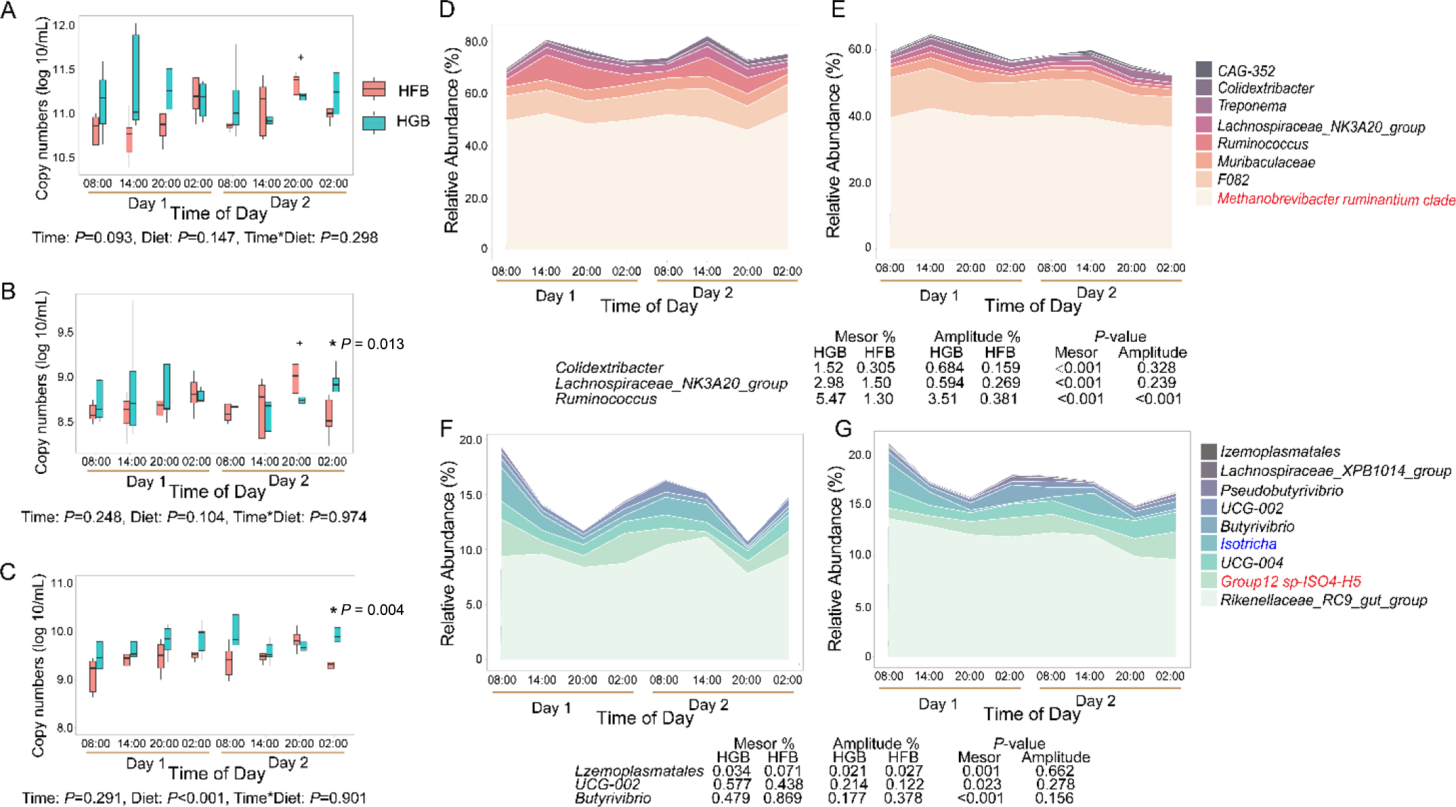
The daily pattern of rumen microbial population and relative abundance of feeding-responsive rumen microbiota. The daily pattern of rumen bacteria (A), archaea (B), and protozoa (C). Statistical inference of pairwise contrast in each time point is indicated by *(*P* < 0.05) and +(0.05 < *P* < 0.10). The daily pattern of FFR microbiota under the HG (D) and HF (E) diet. The daily patterns of SFR microbiota under the HG (F) and HF (G) diet. Fast-feeding-responsive microbiota: the relative abundance of microbial taxa had a significant circadian rhythm under both HG and HF diets and their peak time within the range of 0 to 12 h after feeding. Slow-feeding-responsive microbiota: microbial taxa's relative abundance had a significant circadian rhythm under both HG and HF diets and their peak time within the range of 12 to 24 h after feeding. No significant difference in peak time was observed in the relative abundance of these microbial taxa. The daily patterns of microbial taxa that had a significant difference in mesor or amplitude under different diets are shown on the plot. Mesor: the average level of the diurnal fluctuation. Amplitude: the distance between peak and mesor. Peak time: the time of the peak of rhythm. The legends in black represent the bacterial genus, red represent archaeal species, and blue represent the protozoal genus.

**Figure 3:**
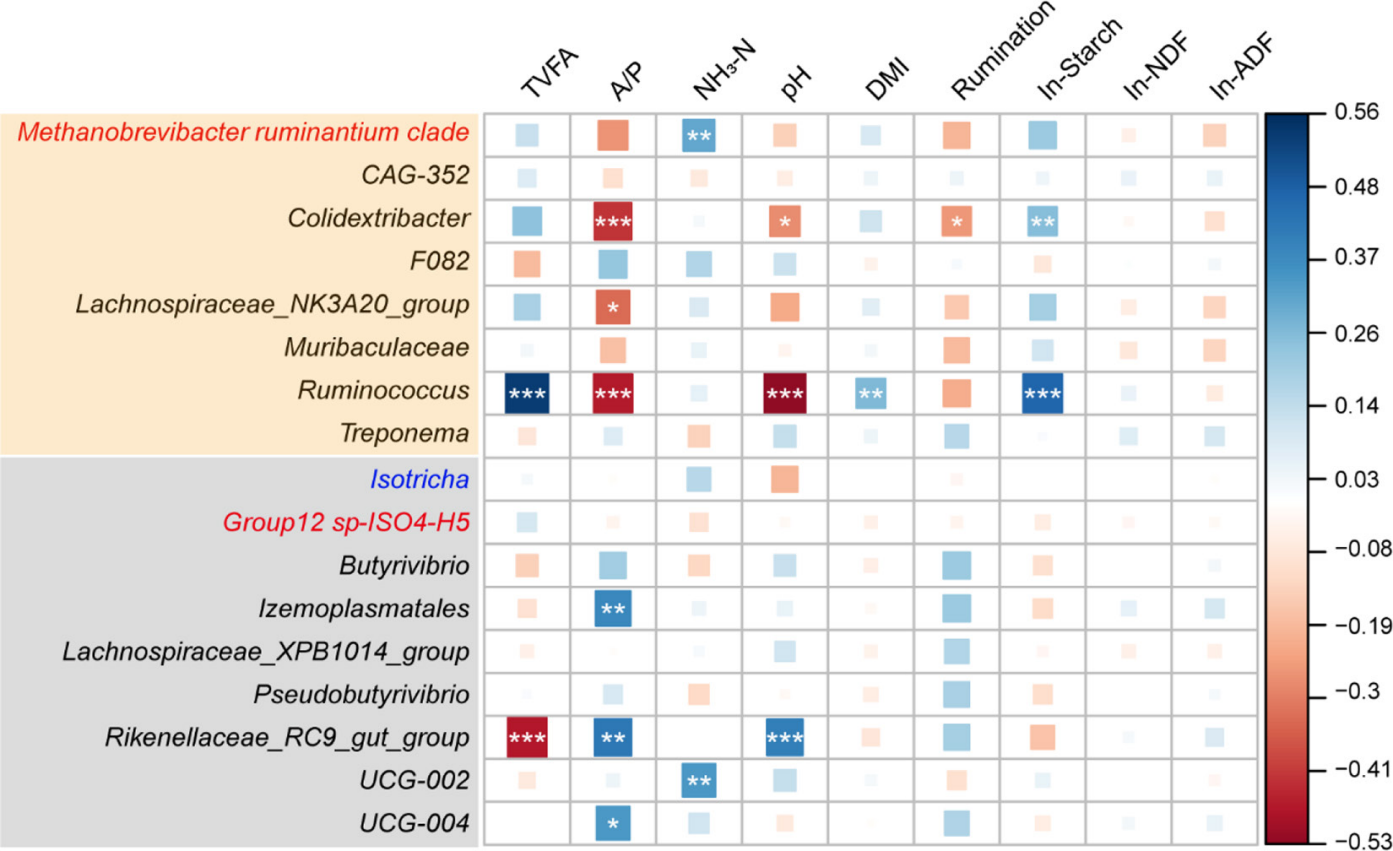
Correlations between rumen microbiota and fermentation profiles, DMI, and rumination. The color denotes the correlation coefficient determined by the linear mixed-effects model. Statistical significance is based on Wald type II χ^2^ tests. The *P*-values were adjusted by false discovery rate: *P < 0.05, **P < 0.01, ***P < 0.001. The microbial taxa in the yellow panel were the FFR taxa whose relative abundance's peak time was in the range of 0 to 12 h after feeding; the microbial taxa in the gray panel were the SFR taxa whose relative abundance's peak time was in the range of 12 to 24 h after feeding. The legends in black represent the bacterial genus, red represent archaeal species, and blue represent the protozoal genus.

Although the driving factors of feeding-time-responsive shift and dynamics are not well defined, one of the mechanisms could be the recently proposed diurnal oscillations of rumen bacteria and archaea (Shaani et al., 2018). Our study further found diurnal oscillations of protozoa in addition to bacteria and archaea with most of them having diurnal oscillations with similar peak time in the rhythm under both HG and HF diets. Nevertheless, in agreement with the previous study, the nutritional niche shifts with the feed intake change throughout the day, which could be the most important factor that leads to these diurnal oscillations in rumen microbiota (Shaani et al., 2018). These diurnal oscillations of rumen microbiota were caused by external factors. The present study also confirmed that the population of rumen bacteria was not affected by diet composition, which was in line with a previous study (Salfer et al., 2021). In addition, the results confirmed that the protozoal population can be affected by diet. Protozoa undergo chemotaxis toward areas with a higher nutrient concentration (Firkins et al., 2020) and starch is a more potent stimulus for protozoal growth than fiber (Arshad et al., 2021). These may cause a higher population of protozoa under the HG diet compared with the HF diet.

In line with a previous study, some rumen microbes had different diurnal oscillation patterns in response to feeding time (Shaani et al., 2018). Also, behavior variation of some of them was related to VFA concentration and fermentation efficiency (A:P ratio), suggesting that manipulation of fast or slow feeding time responders can redirect hydrogen flow. In agreement with the previous study, bacterial taxa (*Lachnospiraceae_NK3A20_group*, *Ruminococcus*, *Muribaculaceae*, *F082*, *CAG-352*, *Treponema*, and *Colidextribacter*) that rapidly ferment nonstructural carbohydrates increased in several hours after feeding regardless of diet composition (Salfer et al., 2021). However, no significant difference was found in the peak time of these rhythmic microbial taxa between the 2 diets. This suggests that individual microbes follow a similar growth pattern under different nutritional environments in the rumen. One reason could be that both HG and HF diets contain starch and nonstructural carbohydrates, which are normally the prioritized substrate to be degraded and fermented in the rumen followed by fibers (Hall and Mertens, 2017). Therefore, the proliferation of individual microbes depends on the available sugar, starch, and fiber. Another reason could be that there are complex dynamics relating to cross-feeding between microbes that affect their growth and ecological niches (Ghoul and Mitri, 2016). This needs to be further studied for the rumen microbes using culturomics to uncover the cross-feeding, competition, or both among these rhythmic microbes. However, previous studies indicate these FFR taxa were more amylolytic, while the SFR taxa were more cellulolytic (Mizrahi et al., 2021; Hua et al., 2022). The observed FFR and SFR taxa could be highly responsive to these nutritional niche shifts and we speculate that manipulating these microbial taxa by their nutritional niches could be a promising approach to enhance rumen fermentation efficiency. Consistent with the study of Ying et al. (2015), rumen fermentation efficiency decreased (A/P ratio increased) with after-feeding hours increasing regardless of the diet composition. This may be due to the increasing of FFR taxa, which led to more propionate and less methane production (Hua et al., 2022). The daily dynamics of FFR and SFR taxa targeting different carbohydrate substrates could be an important contributor to the daily rhythm of A/P ratio (Beckett et al., 2021), which implies that properly modifying the timing of fiber and grain feeding could be a strategy to optimize the microbial cross-feeding to efficient rumen fermentation.

The results in this study indicate the feeding-time-responsive patterns of feed intake, rumination, and rumen fermentation under different diets. Our preliminary results revealed potential different responsive behaviors of rumen microbes. The identified FFR and SFR taxa suggest that the nutritional niche could be the main driving factor contributing to the diurnal oscillations of these microbial taxa, which is further associated with rumen fermentation efficiency (A/P ratio). However, a shorter interval is required to verify our findings and to ascertain which individuals respond rapidly or slowly to feeding times. It is noticeable that the observed change could be due to the confounded factors between the intake and eating behavior of the animals. Additionally, the current experimental design made it impossible to dissect the confounding effects of period and diet on the results. Future studies using cannulated cows with forced feeding in a crossing-over design will help to determine how these factors can play a role in circadian rhythm-driven microbial responses. Regardless, our study has advanced the knowledge of microbial diurnal oscillations in the rumen under different types of diets, which paves the way for targeted approaches to manipulate rumen microbiota.

## References

[bib1] Arshad M.A., Hassan F.U., Rehman M.S., Huws S.A., Cheng Y., Din A.U. (2021). Gut microbiome colonization and development in neonatal ruminants: Strategies, prospects, and opportunities. Anim. Nutr..

[bib2] Bates D., Mächler M., Bolker B., Walker S. (2015). Fitting linear mixed-effects models using lme4. J. Stat. Softw..

[bib3] Beckett L., Gleason C.B., Bedford A., Liebe D., Yohe T.T., Hall M.B., Daniels K.M., White R.R. (2021). Rumen volatile fatty acid molar proportions, rumen epithelial gene expression, and blood metabolite concentration responses to ruminally degradable starch and fiber supplies. J. Dairy Sci..

[bib4] Bolyen E., Rideout J.R., Dillon M.R., Bokulich N.A., Abnet C.C., Al-Ghalith G.A., Alexander H., Alm E.J., Arumugam M., Asnicar F., Bai Y., Bisanz J.E., Bittinger K., Brejnrod A., Brislawn C.J., Brown C.T., Callahan B.J., Caraballo-Rodriguez A.M., Chase J., Cope E.K., Da Silva R., Diener C., Dorrestein P.C., Douglas G.M., Durall D.M., Duvallet C., Edwardson C.F., Ernst M., Estaki M., Fouquier J., Gauglitz J.M., Gibbons S.M., Gibson D.L., Gonzalez A., Gorlick K., Guo J., Hillmann B., Holmes S., Holste H., Huttenhower C., Huttley G.A., Janssen S., Jarmusch A.K., Jiang L., Kaehler B.D., Kang K.B., Keefe C.R., Keim P., Kelley S.T., Knights D., Koester I., Kosciolek T., Kreps J., Langille M.G.I., Lee J., Ley R., Liu Y.X., Loftfield E., Lozupone C., Maher M., Marotz C., Martin B.D., McDonald D., McIver L.J., Melnik A.V., Metcalf J.L., Morgan S.C., Morton J.T., Naimey A.T., Navas-Molina J.A., Nothias L.F., Orchanian S.B., Pearson T., Peoples S.L., Petras D., Preuss M.L., Pruesse E., Rasmussen L.B., Rivers A., Robeson M.S., Rosenthal P., Segata N., Shaffer M., Shiffer A., Sinha R., Song S.J., Spear J.R., Swafford A.D., Thompson L.R., Torres P.J., Trinh P., Tripathi A., Turnbaugh P.J., Ul-Hasan S., van der Hooft J.J.J., Vargas F., Vazquez-Baeza Y., Vogtmann E., von Hippel M., Walters W., Wan Y., Wang M., Warren J., Weber K.C., Williamson C.H.D., Willis A.D., Xu Z.Z., Zaneveld J.R., Zhang Y., Zhu Q., Knight R., Caporaso J.G. (2019). Reproducible, interactive, scalable and extensible microbiome data science using QIIME 2. Nat. Biotechnol..

[bib5] Broderick G.A., Kang J.H. (1980). Automated simultaneous determination of ammonia and total amino acids in ruminal fluid and in vitro media. J. Dairy Sci..

[bib6] Callahan B.J., McMurdie P.J., Rosen M.J., Han A.W., Johnson A.J., Holmes S.P. (2016). DADA2: High-resolution sample inference from Illumina amplicon data. Nat. Methods.

[bib7] Cao Z.J., Li S.L., Xing J.J., Ma M., Wang L.L. (2008). Effects of maize grain and lucerne particle size on ruminal fermentation, digestibility and performance of cows in midlactation. J. Anim. Physiol. Anim. Nutr. (Berl.).

[bib8] Firkins J.L., Yu Z., Park T., Plank J.E. (2020). Extending Burk Dehority's perspectives on the role of ciliate protozoa in the rumen. Front. Microbiol..

[bib9] Ghoul M., Mitri S. (2016). The ecology and evolution of microbial competition. Trends Microbiol..

[bib10] Hall M.B., Mertens D.R. (2017). A 100-year review: Carbohydrates—characterization, digestion, and utilization. J. Dairy Sci..

[bib11] Henderson G., Cox F., Ganesh S., Jonker A., Young W., Janssen P.H., Global Rumen Census Collaborators (2015). Rumen microbial community composition varies with diet and host, but a core microbiome is found across a wide geographical range. Sci. Rep..

[bib12] Hua D., Hendriks W.H., Xiong B., Pellikaan W.F. (2022). Starch and cellulose degradation in the rumen and applications of metagenomics on ruminal microorganisms. Animals (Basel).

[bib13] Mizrahi I., Wallace R.J., Morais S. (2021). The rumen microbiome: Balancing food security and environmental impacts. Nat. Rev. Microbiol..

[bib14] Morais S., Mizrahi I. (2019). Islands in the stream: From individual to communal fiber degradation in the rumen ecosystem. FEMS Microbiol. Rev..

[bib15] Morais S., Mizrahi I. (2019). The road not taken: The rumen microbiome, functional groups, and community states. Trends Microbiol..

[bib16] Newbold C.J., de la Fuente G., Belanche A., Ramos-Morales E., McEwan N.R. (2015). The role of ciliate protozoa in the rumen. Front. Microbiol..

[bib17] Parsons R., Parsons R., Garner N., Oster H., Rawashdeh O. (2020). CircaCompare: A method to estimate and statistically support differences in mesor, amplitude and phase, between circadian rhythms. Bioinformatics.

[bib18] Quast C., Pruesse E., Yilmaz P., Gerken J., Schweer T., Yarza P., Peplies J., Glockner F.O. (2013). The SILVA ribosomal RNA gene database project: Improved data processing and web-based tools. Nucleic Acids Res..

[bib19] Salfer I.J., Crawford C.E., Rottman L.W., Harvatine K.J. (2021). The effects of feeding rations that differ in neutral detergent fiber and starch within a day on the daily pattern of key rumen microbial populations. JDS Commun..

[bib20] Seedorf H., Kittelmann S., Henderson G., Janssen P.H. (2014). RIM-DB: a taxonomic framework for community structure analysis of methanogenic archaea from the rumen and other intestinal environments. PeerJ.

[bib21] Shaani Y., Zehavi T., Eyal S., Miron J., Mizrahi I. (2018). Microbiome niche modification drives diurnal rumen community assembly, overpowering individual variability and diet effects. ISME J..

[bib22] Söllinger A., Tveit A.T., Poulsen M., Noel S.J., Bengtsson M., Bernhardt J., Frydendahl Hellwing A.L., Lund P., Riedel K., Schleper C., Højberg O., Urich T. (2018). Holistic assessment of rumen microbiome dynamics through quantitative metatranscriptomics reveals multifunctional redundancy during key steps of anaerobic feed degradation. mSystems.

[bib23] Stevenson D.M., Weimer P.J. (2007). Dominance of Prevotella and low abundance of classical ruminal bacterial species in the bovine rumen revealed by relative quantification real-time PCR. Appl. Microbiol. Biotechnol..

[bib24] Sylvester J.T., Karnati S.K.R., Yu Z., Morrison M., Firkins J.L. (2004). Development of an assay to quantify rumen ciliate protozoal biomass in cows using real-time PCR. J. Nutr..

[bib25] Thaiss C.A., Zeevi D., Levy M., Zilberman-Schapira G., Suez J., Tengeler A.C., Abramson L., Katz M.N., Korem T., Zmora N., Kuperman Y., Biton I., Gilad S., Harmelin A., Shapiro H., Halpern Z., Segal E., Elinav E. (2014). Transkingdom control of microbiota diurnal oscillations promotes metabolic homeostasis. Cell.

[bib26] Wang H., Xu R., Li Q., Su Y., Zhu W. (2023). Daily fluctuation of colonic microbiome in response to nutrient substrates in a pig model. NPJ Biofilms Microbiomes.

[bib27] Xu J., Wang H., Xu R., Li Q., Su Y., Liu J., Zhu W. (2023). The diurnal fluctuation of colonic antibiotic resistome is correlated with nutrient substrates in a pig model. Sci. Total Environ..

[bib28] Yi S., Dai D., Wu H., Chai S., Liu S., Meng Q., Zhou Z. (2022). Dietary concentrate-to-forage ratio affects rumen bacterial community composition and metabolome of yaks. Front. Nutr..

[bib29] Ying Y., Rottman L.W., Crawford C., Bartell P.A., Harvatine K.J. (2015). The effects of feeding rations that differ in neutral detergent fiber and starch concentration within a day on rumen digesta nutrient concentration, pH, and fermentation products in dairy cows. J. Dairy Sci..

[bib30] Zhou M., Hernandez-Sanabria E., Guan L.L. (2009). Assessment of the microbial ecology of ruminal methanogens in cattle with different feed efficiencies. Appl. Environ. Microbiol..

